# Supplementary material for: DNA methylation reprogramming during seed development and its functional relevance in seed size/weight determination in chickpea
Source: Commun Biol. 2020 Jul 3;3:340. doi: 10.1038/s42003-020-1059-1 (PMC7335156; doi:10.1038/s42003-020-1059-1)
Supplement: Supplementary file 1 — Supplementary Information [file 42003_2020_1059_MOESM1_ESM.pdf]

DNA methylation reprogramming during seed development and its functional relevance in seed size/weight determination in chickpea

Rajkumar et al.

Additional Information

Following Supplementary Material is available for this paper.

- Supplementary Figures 1-18 (*available in this file*)
- Supplementary Tables 1-3 (*available in this file*)
- Supplementary Datasets 1-11 (*available as separate MS Excel files*)

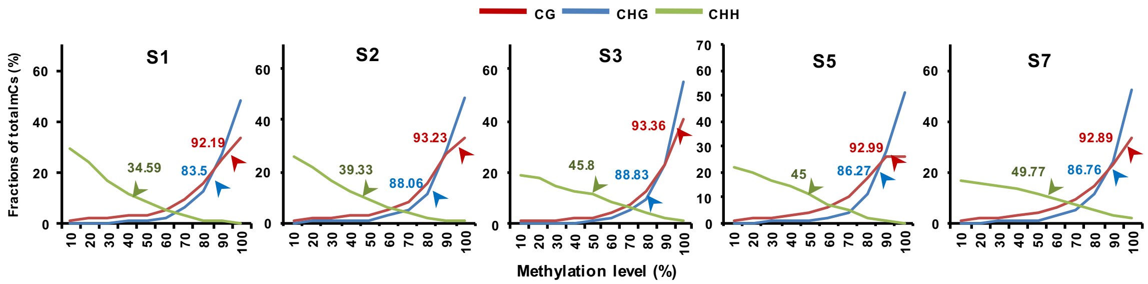

Supplementary Fig. 1. Methylation level in different sequence contexts in JGK 3 cultivar. Fraction of methylcytosines (mCs) exhibiting varying methylation levels in CG, CHG and CHH sequence contexts during seed development stages (S1, S2, S3, S5 and S7) in JGK 3 cultivar is shown. Average methylation level (%) in each sequence context are indicated by arrow heads in different colors at each stage.

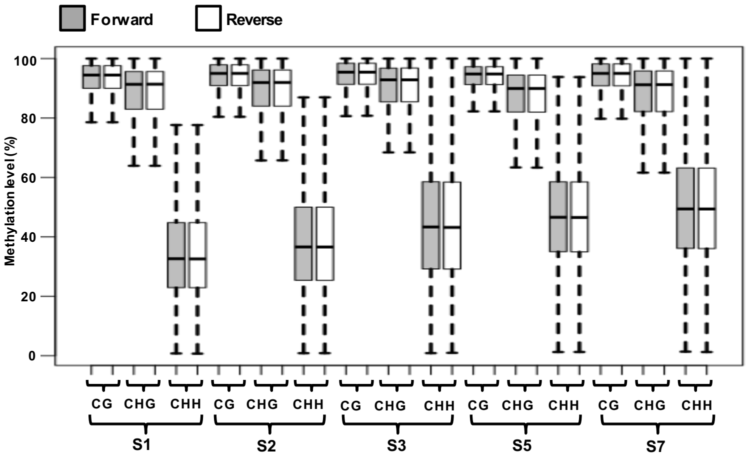

Supplementary Fig. 2. Methylation level in forward and reverse strands. Methylation level in forward and reverse strands in different sequence contexts at different stages of seed development in JGK 3 cultivar is shown via boxplot.

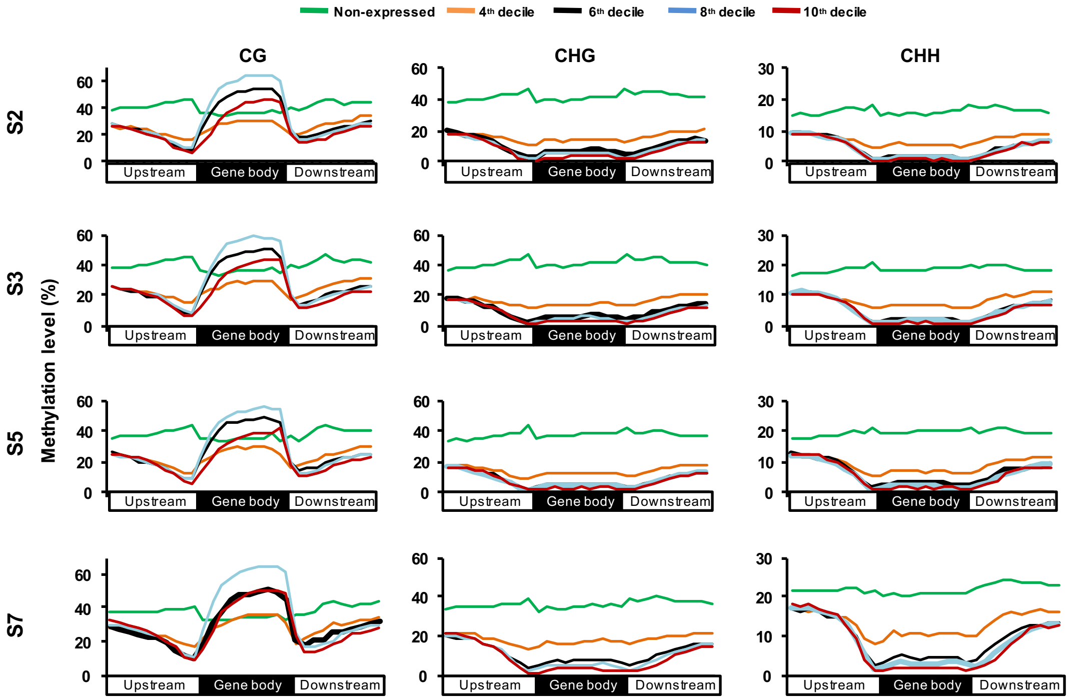

Supplementary Fig. 3. Influence of DNA methylation on gene expression during seed development. Methylation level within gene body and 2 kb flanking (upstream and downstream) regions in different sequence contexts for the gene sets that are expressed at different levels, including non-expressed (2nd decile), low (4th decile), moderate (6th decile), high (8th decile) and highest (10th decile), at S2, S3, S5 and S7 stages is shown. The data for S1 stage of seed development are given in Fig. 1e. Each region was divided into 10 bins of equal size and normalized methylation level for the respective sets of genes in each bin is shown in the line graphs.

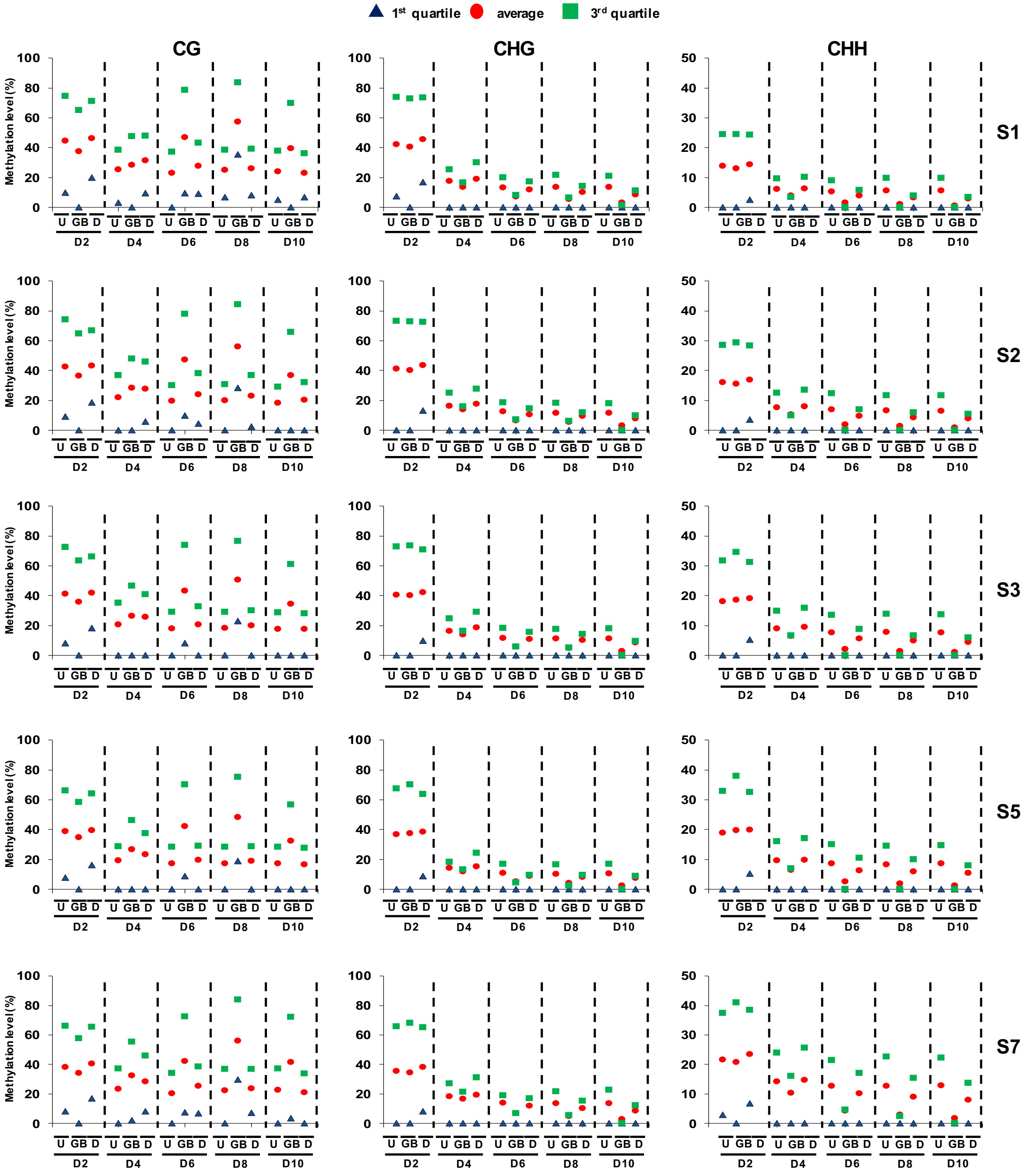

Supplementary Fig. 4. Ist quartile, 3rd quartile and average methylation levels within gene body (GB) and 2 kb flanking (U, upstream and D, downstream) regions in different sequence contexts for the gene sets that are expressed at different levels, including non-expressed (D2, 2nd decile), low (D4, 4th decile), moderate (D6, 6th decile), high (D8, 8th decile) and highest (D10, 10th decile) at different stages of seed development.

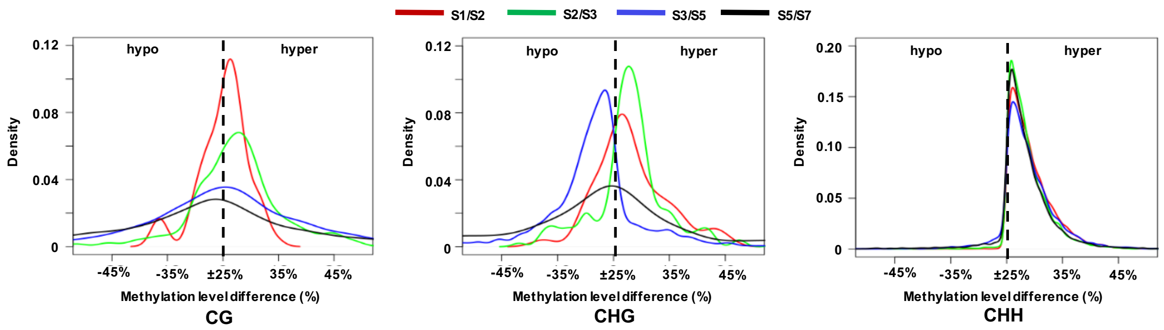

Supplementary Fig. 5. Methylation level difference in CG, CHG and CHH context DMRs between successive stages of seed development is shown via kernel density plots. The DMRs (hypo/hyper) with significant methylation difference (≥25% methylation level difference with <0.01 *q*-value) were analyzed.

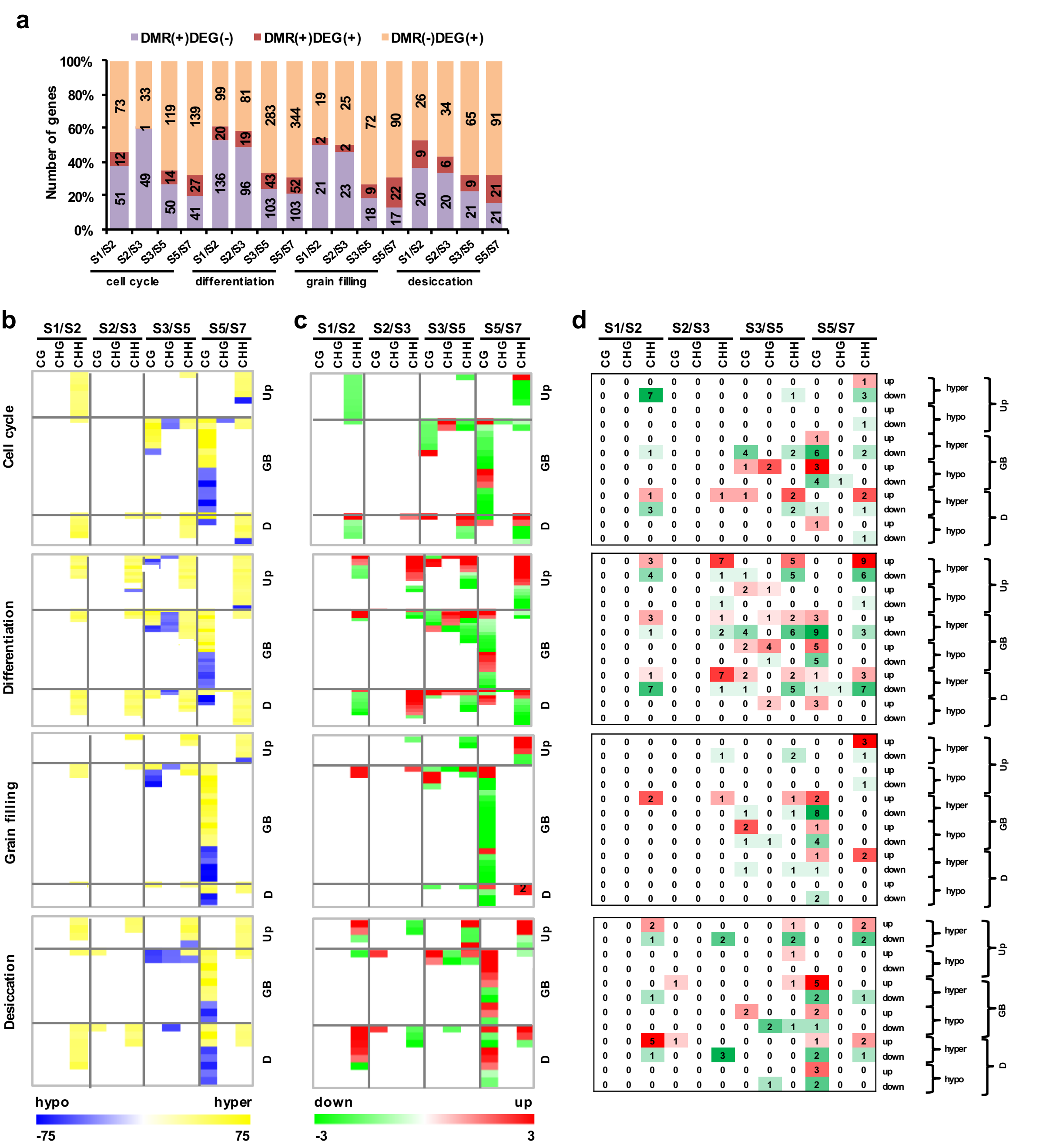

Supplementary Fig. 6. Differential methylation and differential gene expression during seed development for genes involved in important biological processes. (a) Number of DMR-associated with no differential expression [DMR(+)/DEG(-)], DMR-associated differentially expressed [DMR(+)/DEG(+)], and differentially expressed not associated with DMRs [DMR(-)/DEG(+)] genes involved in cell cycle, differentiation, grain filling and desiccation processes is shown in bar graph. (b) Heatmaps showing differential methylation of the genes involved in cell cycle, differentiation, grain filling and desiccation processes in different sequence contexts and genic regions. Scale at the bottom represents percentage of methylation level difference (hypo/hyper). (c) Heatmaps showing differential expression of the differentially methylated genes shown in b. Scale at the bottom represents differential expression (up/down) in $\log_2$ fold-change. (d) Number of differentially expressed (up/down) genes that are differentially methylated (hyper/hypo) in different sequence contexts and genic regions for which heatmaps are shown in b and c. Intensity of red and green colors indicate number of DMR-associated upregulated and downregulated genes, respectively, between successive stages of seed development.

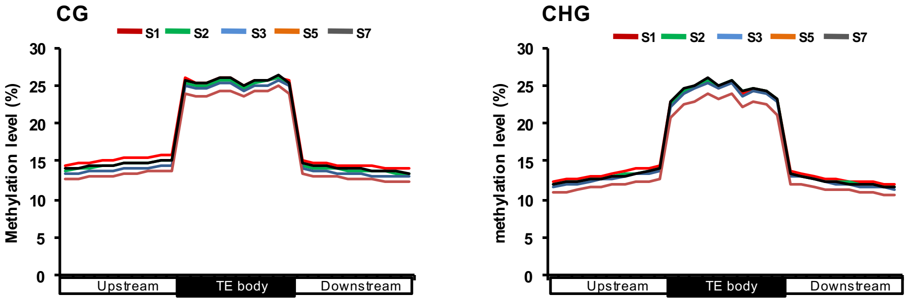

Supplementary Fig. 7. CG and CHG context methylation in transposable elements (TEs) during seed development. Methylation level within TEs and their 2 kb flanking (upstream and downstream) regions in CG and CHG contexts is shown at different stages of seed development. Each region was divided into 10 bins of equal size and normalized methylation level in each bin is shown in the line graphs.

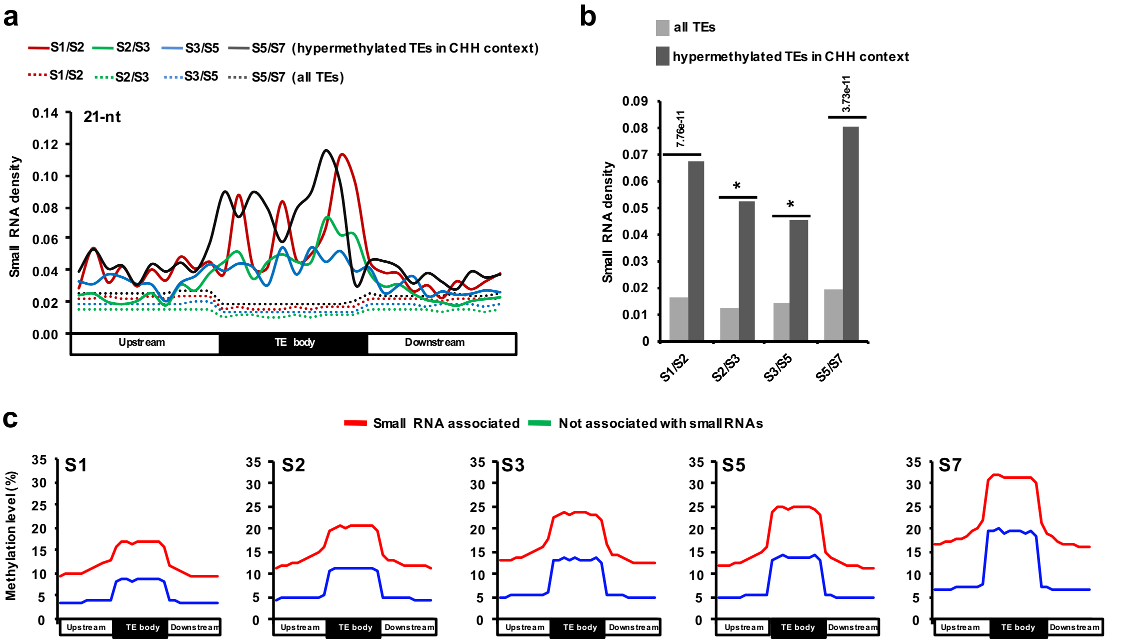

Supplementary Fig. 8. Analysis of 21-nt small RNAs during seed development. (a) Density of 21-nucleotide (nt) small RNAs in hypermethylated TEs in CHH context and all the TEs between successive stages of seed development are shown. Each region was divided into 10 bins of equal size and normalized small RNA density in each bin is shown. (b) Density of 21-nt small RNAs within the TE body of hypermethylated TEs in CHH context and all TEs is shown. The *p*-value of significance as determined by Fisher's exact test is given. Asterisks denote *p*-value <2.2e-16. (c) Methylation level in CHH context within TE body and their 2 kb flanking regions for the TEs associated with 21-nt small RNAs and not associated with small RNAs at different stages of seed development is shown. Each region was divided into 10 bins of equal size and normalized methylation level in each bin is shown.

a

	CG		CHG		CHH	
	U/GB	D/GB	U/GB	D/GB	U/GB	D/GB
S1/S2	0.468	0.374	0.143	0.355	2.2E-16	1.42E-15
S2/S3	0.492	0.469	0.039	0.006	6.387E-09	9.246E-07
S3/S5	0.658	0.221	0.010	1.966E-06	1.669E-05	9.30E-06
S5/S7	0.023	1.80E-04	0.010	2.30E-05	2.758E-06	1.80E-06

b

	CG		CHG		CHH	
	U/GB	D/GB	U/GB	D/GB	U/GB	D/GB
S1/S2	-	-	-	-	0.002	0.002
S2/S3	0.058	0.239	0.390	-	0.662	0.817
S3/S5	0.919	0.823	0.167	7.40E-05	0.005	0.006
S5/S7	0.016	0.001	0.167	8.60E-04	5.00E-04	1.00E-04

Supplementary Fig. 9. Statistical significance of difference in frequency of TEs between gene body and flanking regions in different sets of genes during seed development. (a) *P*-value significance of difference in frequency of TEs between gene body and flanking regions of DMR-associated [DMR(+)] genes in CG, CHG and CHH contexts. Frequency of TEs within gene body and flanking regions of [DMR(+)] genes are shown in Fig. 6d-f. (b) *P*-value significance of difference in frequency of TEs between gene body and flanking regions of differentially expressed genes that are DMR-associated [DMR(+)/DEG(+)] in CG, CHG and CHH contexts. Frequency of TEs within gene body and flanking regions of [DMR(+)/DEG(+)] genes are shown in Fig. 6g-i. *P*-values were determined by Wilcoxon signed rank test. Significant *P*-values (<0.05) are highlighted in yellow color. U, upstream; GB, gene body; D, downstream.

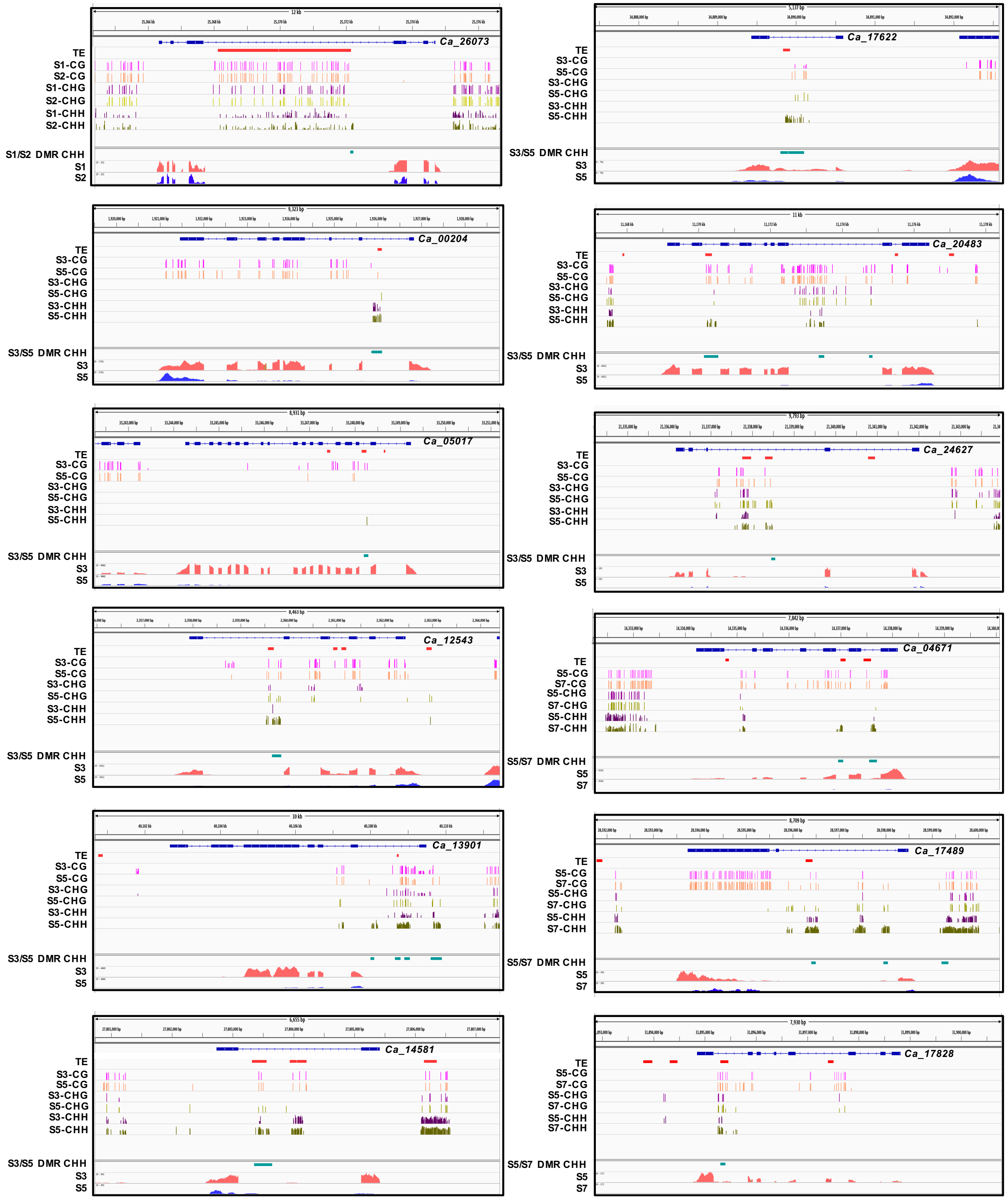

Supplementary Fig. 10. Integrative Genomics Viewer (IGV) view showing differential methylation in different sequence contexts and differential expression of few genes during successive stages of seed development in JGK 3 cultivar. Each IGV plot shows the genomic region corresponding to the gene body and 2 kb flanking regions of the respective gene. The position of transposable element(s) present within gene body and/or flanking regions is also shown. Methylation level of each identified methylcytosine (mC) in the three sequence contexts (CG, CHG and CHH) at the two successive stages is shown in upper six lanes in different colors in each plot. The position of differentially methylated region (DMR) in CHH context between the two stages is also shown. Lower two lanes depicts expression levels of the gene at the two stages of seed development using RNA-seq data.

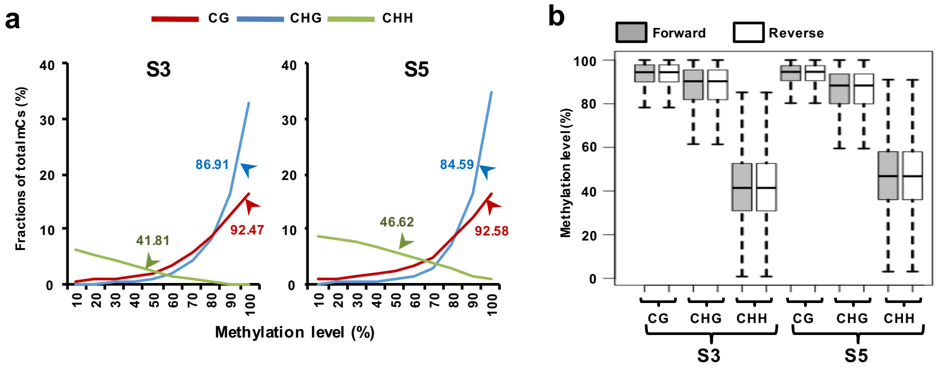

Supplementary Fig. 11. Methylation level in different sequence contexts in Himchana 1 (small-seeded) cultivar. Fraction of methylated cytosines exhibiting varying methylation levels in CG, CHG and CHH sequence contexts at S3 and S5 stages is shown. Average methylation levels in each sequence context are indicated by arrow heads. (b) Methylation level in forward and reverse strands in different sequence contexts at S3 and S5 stages of seed development is shown via boxplot.

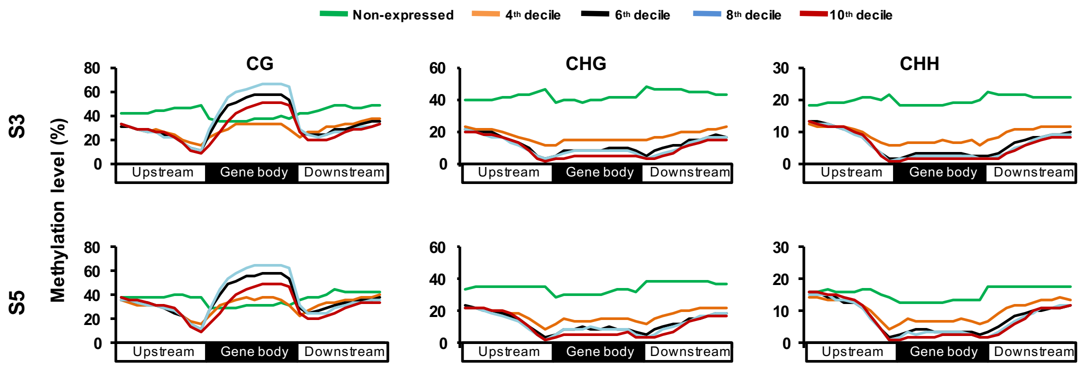

Supplementary Fig. 12. Influence of DNA methylation on gene expression at S3 and S5 stages in Himchana 1. Methylation level within gene body and 2 kb flanking (upstream and downstream) regions in different sequence contexts for the gene sets that are expressed at different levels, including non-expressed (2nd decile), low (4th decile), moderate (6th decile), high (8th decile) and highest (10th decile), at S3 and S5 stages is shown. Each region was divided into 10 bins of equal size and normalized methylation level for the respective set of genes in each bin is shown in the line graphs.

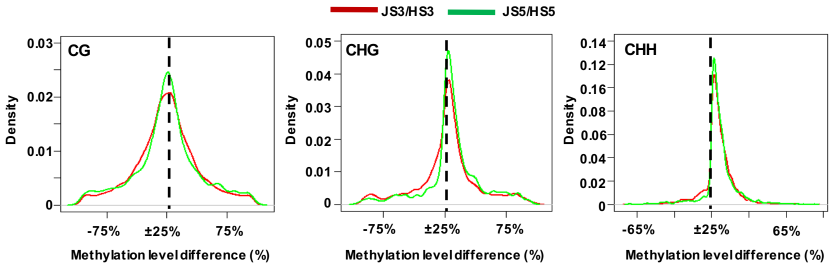

Supplementary Fig. 13. Methylation level difference between JGK 3 and Himchana 1 in different sequence contexts for both stages of seed development is shown via kernel density plots. The DMRs (hypo/hyper) with significant methylation difference (≥25% methylation level difference with <0.01 *q*-value) were analyzed.

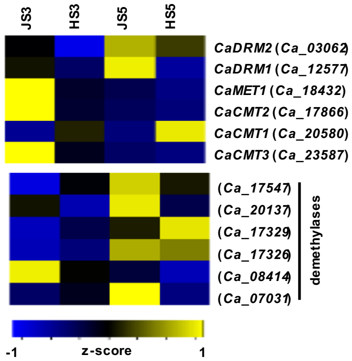

Supplementary Fig. 14. Heatmap showing expression profiles of genes encoding DNA methyltransferases and demethylases at both stages of seed development in the two cultivars. Scale represents z-score based on FPKM values.

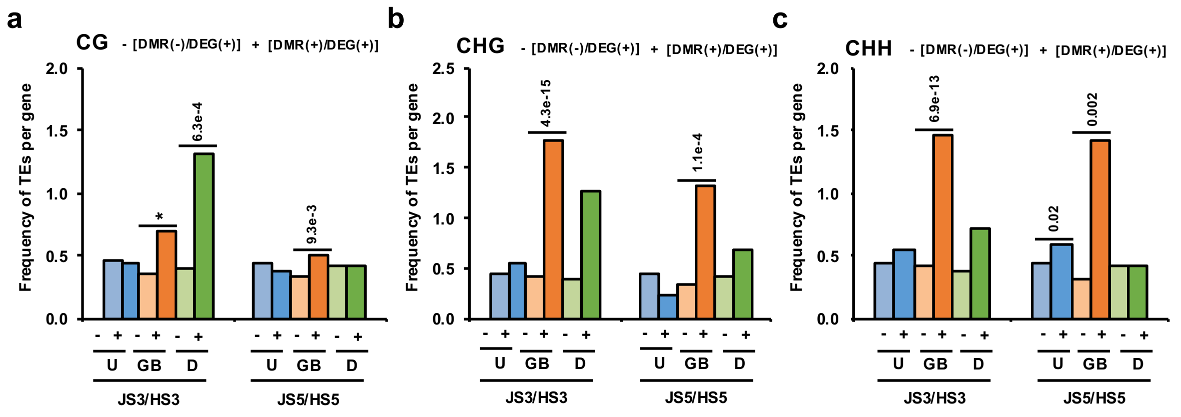

Supplementary Fig. 15. Influence of transposable elements (TEs) on differential gene expression between JGK 3 and Himchana 1 chickpea cultivars at S3 and S5 stages. (a-c) Frequency of TEs within gene body and flanking regions of differentially expressed genes that are DMR-associated [DMR(+)/DEG(+)] and not associated with DMRs [DMR(-)/DEG(+)] in CG (a), CHG (b) and CHH (c) contexts is shown in bar graphs. Significance of difference (*p*-value) was determined by Wilcoxon signed rank test and is indicated above the horizontal bars. Asterisk denotes *p*-value <2.2e-16. U, upstream; GB, gene body; D, downstream.

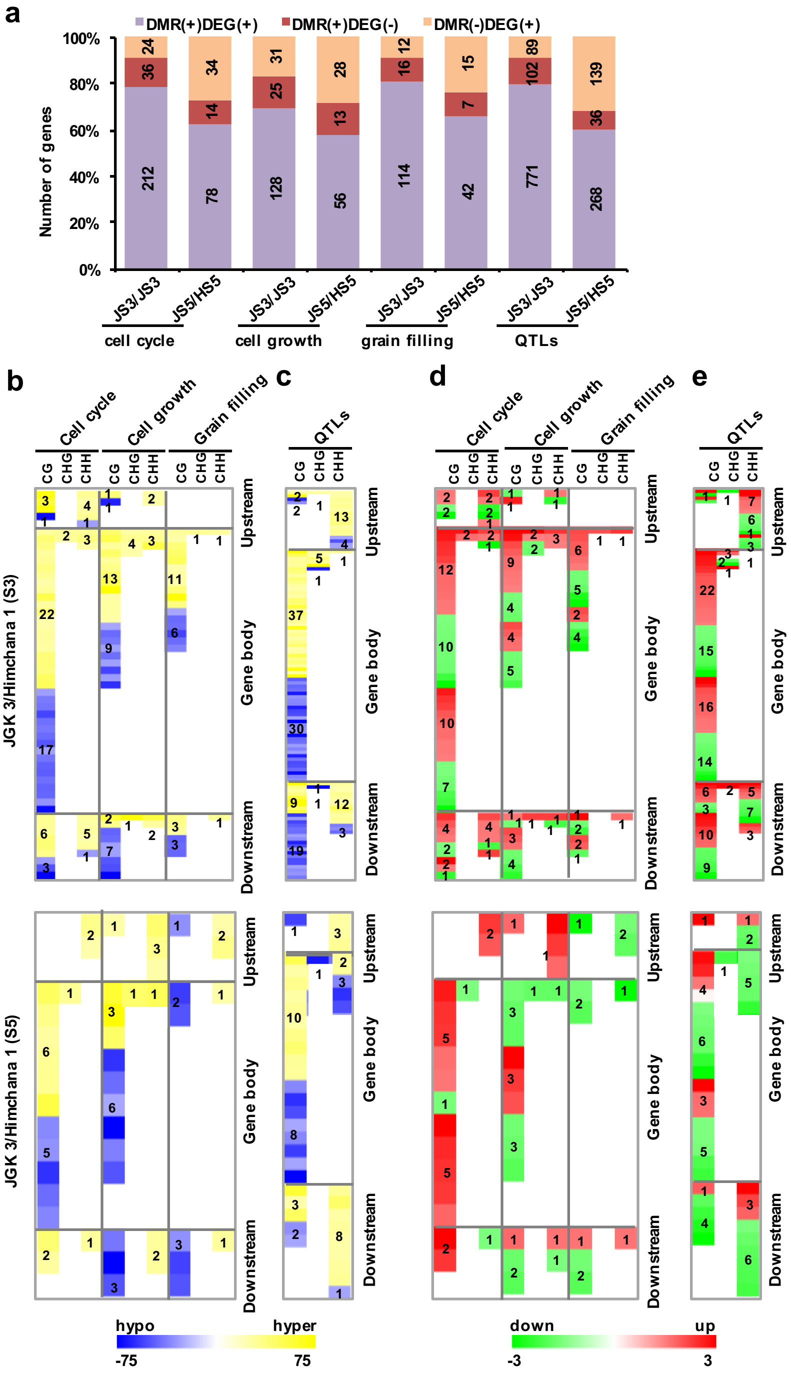

Supplementary Fig. 16. Differential methylation and differential gene expression between JGK 3 and Himchana 1 chickpea cultivars. (a) Number of DMR-associated with no differential expression [DMR(+)/DEG(-)], DMR-associated differentially expressed [DMR(+)/DEG(+)], and differentially expressed not associated with DMRs [DMR(-)/DEG(+)] genes involved in cell cycle, cell growth, grain filling and QTL associated genes is shown in bar graph. (b, c) Heatmaps showing differential methylation between JGK 3 and Himchana 1 cultivars at S3 stage (upper panel) and S5 stage (lower panel) for sets of genes involved in cell cycle, cell growth and grain filling processes (b) and genes located within known QTLs associated with seed size/weight (c). Number of differentially methylated (hyper/hypo) genes in different sequence contexts and genic regions are given. Scale at the bottom represent percentage of methylation level difference. (d, e) Heatmaps showing differential expression between JGK 3 and Himchana 1 cultivars at S3 stage (upper panel) and S5 stage (lower panel) for sets of genes involved in cell cycle, cell growth and grain filling processes (d) and genes located within known QTLs associated with seed size/weight (e). Number of differentially expressed (up/down) genes that are differentially methylated (hyper/hypo) in different sequence contexts and genic regions are given. Scale at the bottom represent $\log_2$ fold-change differential expression.

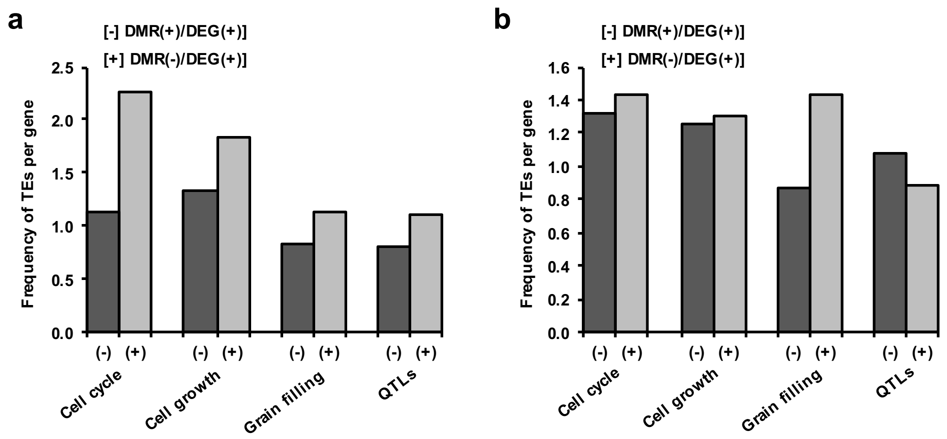

Supplementary Fig. 17. Influence of transposable elements (TEs) on differential gene expression between JGK 3 and Himchana 1 chickpea cultivars. (a, b) Frequency of TEs within gene body and flanking regions of genes involved in cell cycle, cell growth and grain filling processes, and genes located within known QTLs associated with seed size/weight in all the sequence contexts that are differentially expressed between JGK 3 and Himchana 1 at S3 stage (a) and S5 stage (b) of seed development and are DMR-associated [DMR(+)/DEG(+)] or not associated with DMRs [DMR(-)/DEG(+)] is shown in bar graphs. The difference in frequency of TEs was not found to be significant as determined by Wilcoxon signed rank test.

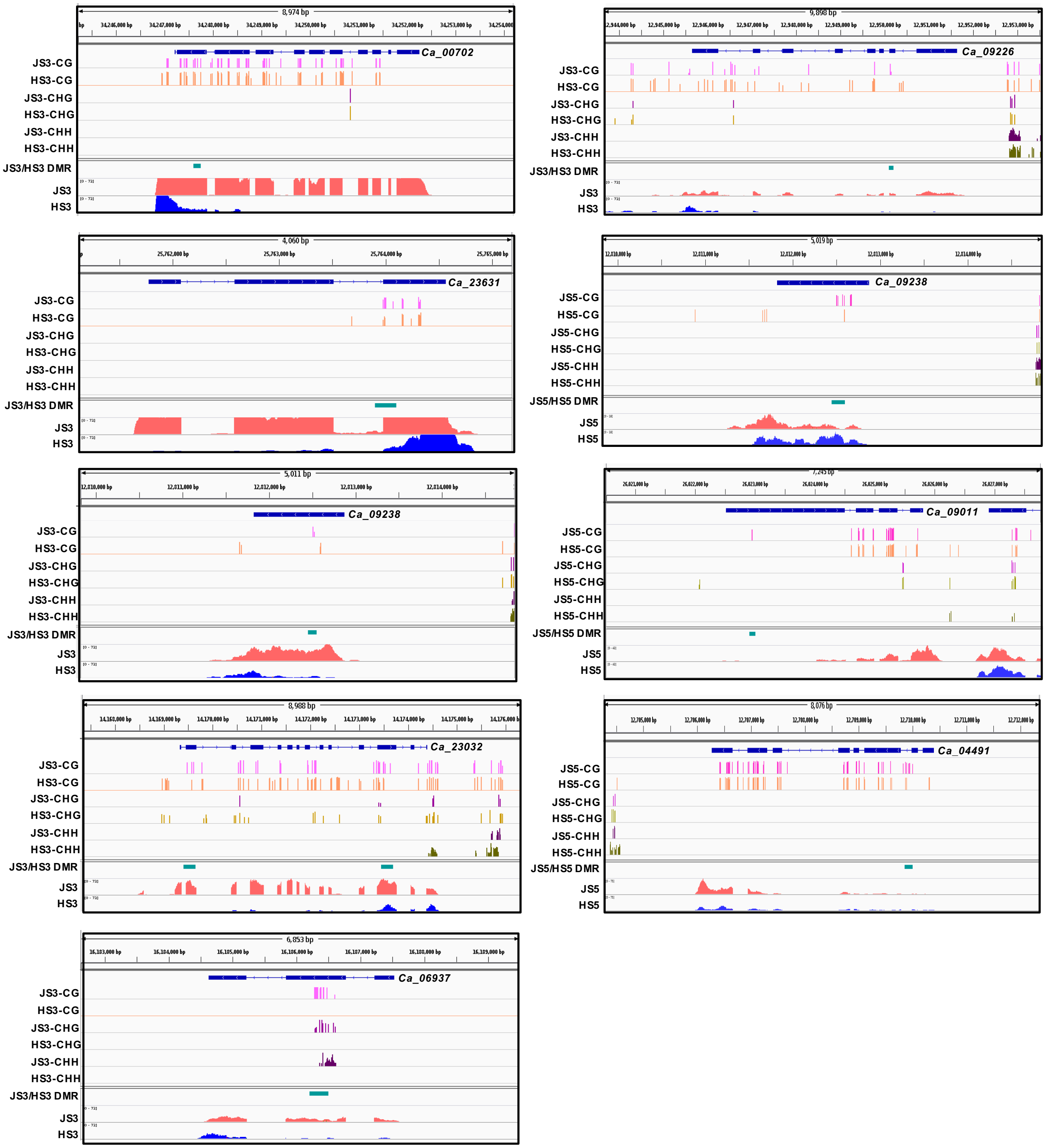

Supplementary Fig. 18. Integrative Genomics Viewer (IGV) view showing differential methylation in different sequence contexts, and differential expression of few genes between JGK 3 and Himchana 1 cultivars at S3 or S5 stage. Each IGV plot shows the genomic region corresponding to the gene body and 2 kb flanking regions of the respective gene. Methylation level of each identified methylcytosine (mC) in the three sequence contexts (CG, CHG and CHH) in both cultivars at S3 (JS3 and HS3) or S5 (JS5 and HS5) stage is shown in upper six lanes in different colors in each plot. The position of differentially methylated region (DMR) in CG context between the two cultivars is also shown. Lower two lanes depicts expression levels of the gene in the two cultivars at S3 or S5 stage using RNA-seq data.

	Replicate(s)	S1	S2	S3	S5	S7
Total read pairs	I	115793788	118500187	117428270	123082547	128725873
	II	122693286	131814052	120168499	122802654	117173008
	III				112454206	
Uniquely mapped read pairs	I	63523237	58624970	55623371	62493026	68526460
	II	65318706	68710205	59583987	59061996	61081477
	III				54224541	
Mapping efficiency (%)	I	54.9	49.5	48.7	52.3	54.6
	II	53.2	52.1	51	49.3	53.4
	III				49.6	
Genome coverage (%)	I	88.91	87.25	87.96	88.66	88.58
	II	88.74	88.21	88.20	88.16	88.07
	III				87.6	
Chloroplast DNA conversion rate (%)	I	99.996	99.994	99.994	99.996	99.995
	II	99.996	99.996	99.995	99.996	99.994
	III				99.995	
Error rate (%)	I	0.004	0.006	0.006	0.004	0.005
	II	0.004	0.004	0.005	0.004	0.006
	III				0.005	
Total methylated cytosines	I	20869394	19784612	21672574	27779745	32471043
	II	20753552	22832746	23117086	25554311	30250903
	III				24074077	
Total methylated cytosines (%)	I	15.91	16.20	17.43	21.42	25.05
	II	15.88	18.05	18.27	20.43	23.86
	III				19.89	
Common methylated cytosines among biological replicates		16808173	16996889	17581361	17859698	25250725

Supplementary Table 1. Summary of bisulphite sequencing data analysis at five successive stages of seed development in JGK 3 chickpea cultivar. S1-S7 represent stages of seed development, including early-embryogenesis (S1), mid-embryogenesis (S2), late-embryogenesis (S3), mid-maturation (S5) and late-maturation (S7).

	21-nt	24-nt
S1	187076	1119112
S2	138346	761710
S3	143303	1219965
S5	381037	2679256
S7	153384	793783

Supplementary Table 2. Number of unique 21-nt and 24-nt small RNAs detected at five successive stages of seed development in JGK 3 chickpea cultivar. These small RNAs were obtained after pre-processing of the raw sequence data via small RNA sequencing as described in the Methods. S1-S7 represent stages of seed development, including early-embryogenesis (S1), mid-embryogenesis (S2), late-embryogenesis (S3), mid-maturation (S5) and late-maturation (S7).

	Replicate(s)	S3	S5
Total read pairs	I	120584177	122478005
	II	122176111	115529146
	III		109535869
Uniquely mapped read pairs	I	63119155	67212217
	II	68619447	69304060
	III		58965287
Mapping efficiency (%)	I	52.3	54.9
	II	56.2	60
	III		53.8
Genome coverage (%)	I	86.59	87.28
	II	87.26	87.2
	III		87.04
Chloroplast DNA conversion rate (%)	I	0.996	0.997
	II	0.997	0.996
	III		0.996
Error rate (%)	I	0.00379	0.00346
	II	0.00323	0.00358
	III		0.0038
Total methylated cytosines	I	24562750	27777737
	II	25784137	26392984
	III		26012659
Total methylated cytosines (%)	I	19.26	21.2
	II	19.68	19.79
	III		20.35
Common methylated cytosines among replicates		19872173	16752284

Supplementary Table 3. Summary of bisulphite sequencing data analysis in Himchana 1 chickpea cultivar. S3 and S5 stages of seed development represent late-embryogenesis and mid-maturation, respectively.
