# Supplementary material for: DNA methylation reprogramming during seed development and its functional relevance in seed size/weight determination in chickpea
Source: Commun Biol. 2020 Jul 3;3:340. doi: 10.1038/s42003-020-1059-1 (PMC7335156; doi:10.1038/s42003-020-1059-1)
Supplement: Supplementary file 14 — Description of Additional Supplementary Files [file 42003_2020_1059_MOESM14_ESM.pdf]

Supplementary Data

Supplementary Dataset 1. Whole genome DNA methylation and its influence on gene expression at different stages of seed development in JGK 3.

Supplementary Dataset 2. Differentially methylated regions in different sequence contexts between successive stages of seed development in JGK 3.

Supplementary Dataset 3. Differential methylation in different sequence contexts and genic regions and differential expression of DMR-associated differentially expressed genes between successive stages of seed development.

Supplementary Dataset 4. List of genes involved in cell cycle, cell growth, grain filling and QTLs that are associated with DMRs and/or show differential expression between JGK 3 and Himchana 1 chickpea cultivars at S3 and S5 stages.

Supplementary Dataset 5. Methylation level within TEs and their flanking regions in different sequence context at different stages of seed development.

Supplementary Dataset 6. Small RNAs mediated methylation in TEs during seed development in JGK 3.

Supplementary Dataset 7. Frequency of TEs within gene body and flanking regions of protein coding genes that are DMR-associated, not associated with DMRs, differentially expressed genes that are DMR-associated and not associated with DMRs in different sequence contexts.

Supplementary Dataset 8. Number of methylcytosines in different sequence contexts in small-seeded (Himchana 1) and large-seeded (JGK 3) cultivars at S3 and S5 stages of seed development.

Supplementary Dataset 9. Differentially methylated regions in different sequence contexts between JGK 3 and Himchana 1 at S3 and S5 stages.

Supplementary Dataset 10. Differential methylation in different sequence contexts and genic regions and differential expression of DMR-associated differentially expressed genes between chickpea cultivars at S3 and S5 stages.

Supplementary Dataset 11. List of genes involved in cell cycle, differentiation, grain filling and desiccation processes that are associated with DMRs and/or show differential expression during successive stages of seed development.
